# Long-Term Assessment of Left Ventricular Ejection Fraction and Mitral Regurgitation Following Takeuchi Repair

**DOI:** 10.21470/1678-9741-2018-0376

**Published:** 2019

**Authors:** Gökmen Akkaya, Çağatay Bilen, Osman Nuri Tuncer, Mehmet Fatih Ayık, Yüksel Atay

**Affiliations:** 1 Department of Cardiovascular Surgery,Ege University School of Medicine, Izmir, Turkey.

**Keywords:** Bland White Garland Syndrome, Mitral Valve Insufficiency, Mitral Valve, Pulmonary Artery, Stroke Volume, Vascular Surgical Procedures, Medical Records

## Abstract

**Objective:**

This study aimed to evaluate the early operative outcomes and to compare the left ventricle and mitral valve functions after initial Takeuchi repair in patients with anomalous left coronary arising from pulmonary artery (ALCAPA).

**Methods:**

Fourteen patients (5 males, 9 females; mean age 4.3 years, ranging from 25 days to 34 years) who were operated for ALCAPA between 2007 and 2018 were included in this study. Data were evaluated retrospectively based on our medical records.

**Results:**

Hospital mortality rate was 7.1% (n=1). Thirteen surviving patients were kept in follow-up mean 4.3±3.05 years. When compared to preoperative measurements, both left ventricular ejection fraction (LVEF), (*P*=0.007) and mitral regurgitation (MR) (*P*=0.001) significantly improved before discharge. Moreover, LVEF values were improved in the late follow-up, considering early postoperative outcomes, and this alteration was significant (*P*=0.014). Nevertheless, alteration in the degree of MR among patients did not differ in the long-term follow-up (*P*=0.180). There was no late-term mortality or need for reoperation among patients.

**Conclusion:**

Although some centers prefer to direct implantation in ALCAPA, Takeuchi procedure can be accepted as a reliable method that provides satisfactory long-term results, considering that it aids to improve left ventricle ejection fraction and reduced mitral valve regurgitation.

**Table:** 

Abbreviations, acronyms & symbols	
ALCAPA	= Anomalous left coronary arising from pulmonary artery
ASD	= Atrial septal defect
CPB	= Cardiopulmonary bypass
ECMO	= Extracorporeal membrane oxygenation
LAD	= Left anterior descending
LVEF	= Left ventricular ejection fraction
MR	= Mitral regurgitation
MRG	= Myocardial revascularization group
NYHA	= New York Heart Association
PDA	= Patent ductus arteriosus
SD	= Standard deviation
TTE	= Transthoracic echocardiography

## INTRODUCTION

Anomalous left coronary artery arising from the pulmonary artery (ALCAPA), also known as Bland-White-Garland syndrome, is a very rare congenital coronary disorder that occurs in approximately 1 in 300,000 live births^[1,2]^. Although when left untreated mortality rate may up to 90% within the first year of life, in the presence of sufficient intercoronary collateral flow, some cases survive even asymptomatic until adulthood^[3]^. Nevertheless, such cases are in danger of sudden death, therefore as soon as the diagnosis is confirmed, regardless of age or clinical manifestation, surgical treatment establishing two coronary systems should be undertaken immediately^[4]^.

In recent years, with the help of advanced techniques and improved intensive care management, surgical correction provides satisfactory results and uneventful survival rate^[5-7]^. However, beyond the lack of comparison of the different well-described surgical techniques in large cohorts, some other issues are still controversial and remain challenging, particularly on the basis of recovery rates in reduced left ventricular function and degree of regression in mitral valve incompetency in long-term follow-up^[6,7]^. In this study, we aimed to present our surgical outcomes and to assess the postoperative recovery in cardiac functions.

## METHODS

Overall, 14 patients who were diagnosed with ALCAPA syndrome and underwent surgical correction in our institute over the period from 2007 to 2018 were included in this study. Hospital records were collected from an electronic database and analyzed retrospectively. Data included preoperative details, surgical process, operative management, perioperative course, postoperative outcomes and echocardiographic evaluation in regular cardiology follow-up. This study was conducted with the approval of local institutional ethics committee and adhered to the principles of the Declaration of Helsinki. A written consent form was obtained from all patients or each parents of children.

### Demographics and Patient Data

The 14 patients (5 male, 9 female) were diagnosed with ALCAPA during the study period. The median age was 4.3 years (ranging from 25 days to 34 years). Eleven of them (78.5%) were admitted to our clinic in childhood. Age at late presentation was 8, 10 and 34 years, respectively. The clinical presentation in the admission time to the hospital was varied. Most children were suffering from episodic sweating, dyspnea or poor feeding and other clinical features of cardiac failure. Beyond childhood two patients (14.2%) presented palpitation and one patient (7.1%) had an angina as well. Meanwhile, dilated cardiomyopathy was observed in six patients (42.8%). In five patients, preoperative inotropic support was needed; furthermore, three of these patients had to be intubated before the surgical process.

Left ventricular function was assessed by transthoracic echocardiography (TTE) with left ventricular ejection fraction (LVEF). Mean LVEF was calculated as 37.14±13.25. The degree of mitral regurgitation (MR) was graded from 0 to 4 (0=none; 1=trivial; 2=mild; 3=moderate; 4=severe) on color Doppler ultrasonography. All cases were imaged via computed tomography aiming to confirm the diagnosis of ALCAPA and to identify the associated lesions. Overall, nine patients (64.2%) had trivial or mild MR besides five of them (35.7%) were at level 3 or 4 (Table 1).

**Table 1:** Preoperative characteristics of patients.

	Age/sex	Weight (kg)	Major symptoms at presentation	LVEF	Preoperative MR	Inotropic support	Need of intubation	Concomitant anomaly
1	4m/F	4	Poor feeding	35	‘1-2	-	-	ASD
2	2m/F	4.5	Dyspnea, sweating	25	‘2	-	-	ASD
3	1y/M	8.5	Rapid breathing	60	‘1	-	-	-
4	6m/M	5	Failure to thrive	30	‘3	+	-	-
5	7m/F	9	Dyspnea, sweating	25	‘2	+	+	-
6	6m/F	6.5	Tachypnea, sweating	30	‘3-4	+	+	-
7	10y/F	28	Palpitation	20	‘3	+	+	-
8	8y/M	22	Incidental murmur	50	‘2	-	-	LAD-RV fistula
9	5m/M	4	Failure to thrive, dyspnea	40	‘3	-	-	-
10	1y/F	8	Failure to thrive	45	‘3-4	-	-	-
11	4m/F	6	Dyspnea, sweating	40	‘1-2	-	-	-
12	1m/F	3.5	Dyspnea, poor feeding	20	‘2	+	-	-
13	34y/F	54	Angina, palpitation	60	‘1	-	-	-
14	25d/F	4	Dyspnea, sweating	40	‘2	-	-	ASD/PDA

ASD=atrial septal defect; LVEF=left ventricle ejection fraction; MR=mitral regurgitation; PDA=patent ductus arteriosus

Cardiac catheterization was applied to four patients in an attempt to visualize the collateral flow in our clinic, in addition to one adult case having previously been examined in another center, while examining for the chest pain. Therewithal, three patients had concomitant atrial septal defect (ASD) and one of those also had patent ductus arteriosus (PDA). In one case, there was an associated fistula between left anterior descending (LAD) coronary artery and the right ventricle associating a stenosis in the proximal left anterior descending coronary artery.

### Surgical Technique

We prefer to create an intrapulmonary tunnel with autologous pericardium, differently from the usual Takeuchi procedure, aiming to reduce the aneurysm development in advanced years. In the surgical process, mediastinal access was achieved through median sternotomy. Cardiopulmonary bypass (CPB) was established following ascending aorta and bicaval cannulation with venting through the right superior pulmonary vein. Soon after conducting CPB, both pulmonary artery branches were snared to promote antegrade perfusion in ALCAPA. After cross-clamp application, antegrade cardioplegia (30 ml/kg, Buckberg solution) is delivered through the ascending aorta. A transverse incision was performed on the surface of main pulmonary artery and the maintenance dose of cardioplegia solution is administered through left coronary orifice. An aortopulmonary window was constructed and a tunnel manufactured from fresh autologous pericardium was created, encircling the left pulmonary ostium throughout the pulmonary artery in a way to maintain the arterial flow from the ascending aorta towards the left coronary orifice. Later on, another pericardial patch was constituted by tempering to close the defect on the upper surface of the pulmonary artery.

Concurrently, ASD closure was utilized in three patients and one of them had a concomitant PDA ligation in the same session. Only in one case, in the presence of a fistula between right ventricle and left anterior descending (LAD) coronary artery, in addition to Takeuchi procedure, the left intermammary artery was anastomosed to LAD following the ligation of the fistula orifice. Thus, LAD and circumflex coronary artery flows were supplied separately.

### Statistical Analysis

Statistical analysis was performed using the IBM SPSS Statistics version 20.0 software (SPSS Inc., Chicago, IL, USA). Descriptive data for continuous variables were reported as mean ± standard deviation (SD), median, number (n), and frequency (%). A nonparametric test (Wilcoxon signed rank test) was used to analyze differences between pre- and postoperative outcomes due to the small sample size, rather than a parametric test. A value of *P*<0.5 was considered statistically significant.

## RESULTS

Hospital mortality was seen in only one patient (7,1%). The death occurred due to the presence of severe heart failure and respiratory distress following prolonged mechanical ventilatory support at the day 14 after surgical correction. This patient was a 10-year-old female patient with a significant ventricular dysfunction (LVEF 20%) and severe mitral valve regurgitation requiring intubation and inotropic support preoperatively. The patient could be weaned from CPB with inotropic support. In the 1^st^ postoperative day, she was examined via TTE and computed tomography to evaluate the left coronary flow. There was no thrombosis detected or tunnel obstruction that could reveal the cause of non-recovered ventricular dysfunction. However, the reduced LVEF value was remaining. Thereafter, mortality due to accompanying acute kidney injury and pneumonia during prolonged intensive care unit stay was observed.

The mean time of extubation and intensive care unit stay were 2.5 (range 1-14), 3.6^[1-14]^ days, respectively. Length of hospital stay time was calculated as 9.5^[5-14]^ days. None of our patients required a ventricular assist device or extracorporeal membrane oxygenation (ECMO) support on presentation or postoperatively. Postoperative mean follow-up time was 4.3±3.05 years. Operative values, intensive care parameters and length of hospital stay were indicated in Table 2.

**Table 2:** Operative results and postoperative outcomes.

Characteristics	Valid (n=13)
CPB time, minutes	100.7±23.86
Aortic cross-clamp time, minutes	85.18±19.36
Mechanical ventilation, days	2.5 (1-14)
ICU length of stay, days	3.6 (1-14)
Hospital length of stay, days	9.5 (5-14)
Hospital survival	13 (92.8%)
Follow-up time, years	4.3±3.05
Mitral valve intervention	0
Coronary artery bypass	1
Septal defect closure	3
PDA closure	1
Left ventricular ejection fraction	57± 6
Residual mitral regurgitation	5
Trivial	3
Mild	2
Moderate	0
Severe	0

Values are mean, SD or n (%). CPB=cardiopulmonary bypass; ICU=intensive care unit; PDA=patent ductus arteriosus

### Follow-Up

The mean duration of postoperative follow-up was 52 months (range 3-120). All patients survive at the time of the recent follow-up, without any complication requiring reoperation. Beyond that, all patients were in New York Heart Association (NYHA) class I-II. All patients were examined via TTE in the first and third month after the surgery, then kept in follow-up of annual TTE imaging.

**Fig. 1:**
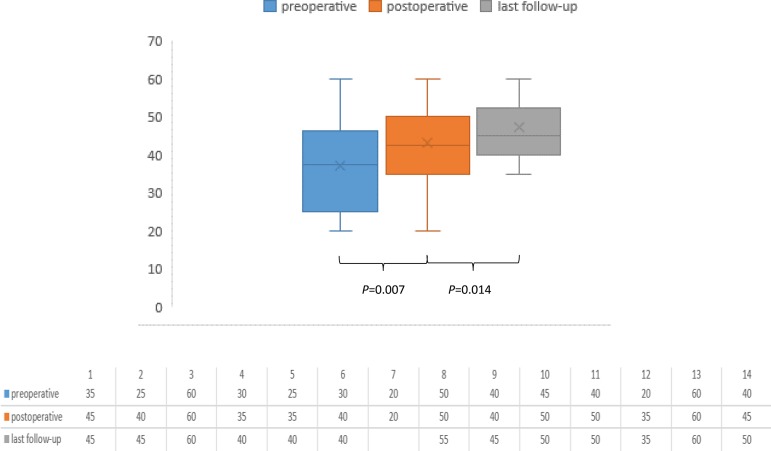
Left ventricle ejection fraction values.

**Fig. 2:**
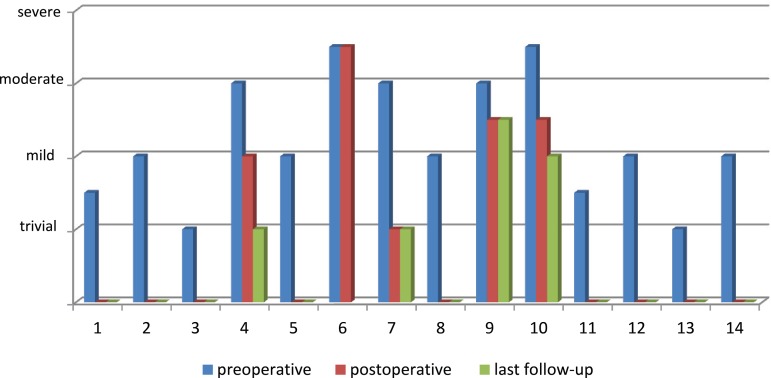
Degrees of mitral regurgitation measurements.

TTE examinations performed in the 1^st^ postoperative week prior to discharge revealed significantly improved LVEF values (*P*=0.007) (Figure 1). Moreover, the degree of MR was also significantly reduced (*P*=0.001), (Figure 2). LVEF values were observed as significantly increased considering early postoperative measurements (*P*=0.014).

Although the majority of patients were totally asymptomatic, two cases went on suffering exertional dyspnea even less severe. Moderate mitral regurgitation was revealed in these cases, therefore dual therapy with spironolactone and angiotensin-converting enzyme inhibitor was administered to these. Ventricular septal thickness and posterior wall thickness were observed as increased following surgery in all patients. However, in subsequent examinations, three cases with septal over-hypertrophy were reduced, afterwards all patients had acceptable left ventricular function in further examinations. Trivial or moderate regurgitation in nine patients was completely cured, therewithal the degree of mitral regurgitation was decreased in all thirteen patients following surgery. Although the comparison between pre- and postoperative MR levels was significant among patients, these postoperative values were not statistically different from late-term outcomes (*P*=0.180).

## DISCUSSION

ALCAPA causes a chronically depressed, ischemic and hypocontractile myocardium due to the supply of hypoxic blood flow towards the left coronary system. Over time, subendocardial necrosis develops and irreversible changes occur. These changes lead to reduced left ventricular and mitral valve functions. In this study, we evaluated the postoperative changes in these issues and assessed the healing process development in the late follow-up.

In the presence of coexisting collateral circulation between the right and left coronary arteries, this progress may be decelerated, thus, the symptoms manifest beyond childhood^[3-8]^ In addition, some patients may be kept on follow-up with misdiagnosis. Particularly dilated cardiomyopathy often delays the accurate diagnosis, as well as four patients in our case serial^[4,7-10]^. Therefore, we are in consensus with Sarıoglu et al.^[7]^ that in every child diagnosed with dilated cardiomyopathy, ALCAPA must be excluded. On the other hand, our oldest patient was a 34-year-old woman suffering from angina and misdiagnosed, thereby imaging extensive blood flow supposed as ventricular septal defect.

Nevertheless, in the case of developing chronic subendocardial fibrosis, sudden death risk remains even after surgical correction^[9]^. Therefore, restoring a dual coronary system is essential. In past decades, several surgical techniques have been described for this purpose.

Since the initial attempt by Sabiston et al.^[11]^ in 1960, the surgery for ALCAPA evolved in advanced years. Nowadays, direct coronary translocation which is first introduced by Niches et al.^[12]^ and Takeuchi^[13]^ methods is frequently used as operative choice. The Takeuchi method is composed of the creation of an aortopulmonary window and the reconstruction of an intrapulmonary tunnel that connects the aorta and the left coronary orifice. Especially the latter is feasible in most patients, even when aortic reimplantation is not possible. Although there was no significant complication rates between the two, both methods require caution and include own potential risks^[14]^. For instance, a baffle leak and supravalvular pulmonary artery stenosis in late-term or a damage to aortic valve while creating the aortopulmonary window are some of theme^[7,9,14-16]^ To avoid these complications, modifications of Takeuchi repair are described. Various materials such as autologous pericardium, a subclavian segment or polytetrafluoroethylene (Gore-Tex) graft options are practiced to construct a tunnel so far^[17-19]^. However, extrapulmonary tunnel construction is also favored by some surgeons^[7]^. Considering all, we always use Takeuchi repair and favor the creation of an intrapulmonary baffle to recover the pulmonary artery via fresh pericardium. As a consequence of these precautions, we had no late complications requiring reoperation yet.

Postoperative mortality varies from 0% to 16% in the literature^[14]^. We observed only one (7.1%) mortality due to cardiac failure and prolonged intubation. This patient was substantially ill, with poor LVEF and severe MR, in addition to receiving inotropic and ventilatory support before the operation. Although reduced LVEF and severe MR are previously associated with mortality, due to the insufficient population of patients we are not able to identify the mortality factors. However, there are reports with successful outcomes, indicating that patients with severe MR and ventricular dysfunction may benefit from ECMO support^[14,20]^.

The increase in LVEF is rapidly developed and reached normal values during the first year in most of our patients. Although we have not used it, fractional shortening measurement is also a beneficial tool to evaluate the ventricle functions and correspond to the LVEF values^[7]^. Consistent with previous articles, we observed a faster recovery in ventricular functions following ALCAPA repair in early ages. Stern et al.^[21]^ confirmed these results by demonstrating perfusion defects on thallium 201 imaging in patients who underwent ALCAPA correction at an elder age. Therewithal, Ben Ali et al.^[22]^ also revealed, with MRG images, scattered subendocardial fibrotic areas, contrary to the improvement on global ventricular functions in TTE examinations.

In MR etiology, left ventricle dilation leading to mitral annular enlargement and ischemic dysfunction of the papillary muscles play an important role. Hence, proper revascularization contributes to reduce MR severity. Therefore, numerous institutes do not address a mitral valve approach simultaneously^[6,7,9,22-24]^. Similarly to us, Neumann et al.^[14]^ globally determined 4 patients with postoperative moderate MR, of which 50% have moderate or severe MR before operation. Moreover, as in LVEF assessment, the result of MR after surgery was better among younger patients. These evidences may be interpreted in countenance of the excellent regenerative potential of the immature myocardium. Nonetheless, the presence of severe MR at presentation in patients with preserved left ventricular function, especially after childhood, may require a concomitant mitral valve repair. Though, we have not experienced such a case yet, we agree with the authors who suggest evaluating the MR profoundly in each patient^[23,24]^. 

Another issue worthy of attention regarding this healing process is a rapidly developed left ventricular and septal hypertrophy. We had two cases with a remarkable septal hypertrophy in TTE before discharge. However, this hypertrophy regressed over time and subsequent examinations were uneventful. Some researchers who observed this compensatory hypertrophy hold the hibernate myocytes responsible for this subject^[7,15,18]^.

This study contains the limitations of its intrinsic retrospective design with the inherent restrictions. The small sample size also causes limitations and hinders to determine further analysis. Thereby we utilize a single technique to all patients; a comparison among various surgical approaches is not possible. When the incidence of ALCAPA is considered, these issues can only be overcome by long-term based on multicentre studies.

## CONCLUSION

In conclusion, despite ALCAPA is a rare entity, early diagnosis is essential and surgical correction should be performed prior to the occurrence of irreversible changes in the myocardium. Therefore, ALCAPA should always be thought in diagnosis, especially in children with dilated cardiomyopathy. In the early postoperative period, LVEF increases significantly and the MR recovers substantially, furthermore these healing processes also continue in the late term. Takeuchi repair in ALCAPA is a feasible method that provides satisfactory results with low mortality and negligible complication rates.

**Table:** 

Authors’ roles & responsibilities	
GA	Substantial contributions to the conception or design of the work; or the acquisition, analysis, or interpretation of data for the work; agreement to be accountable for all aspects of the work in ensuring that questions related to the accuracy or integrity of any part of the work are appropriately investigated and resolved; final approval of the version to be published
ÇB	Substantial contributions to the conception or design of the work; or the acquisition, analysis, or interpretation of data for the work; drafting the work or revising it critically for important intellectual content; final approval of the version to be published
ONT	Substantial contributions to the conception or design of the work; or the acquisition, analysis, or interpretation of data for the work; final approval of the version to be published
MFA	Agreement to be accountable for all aspects of the work in ensuring that questions related to the accuracy or integrity of any part of the work are appropriately investigated and resolved; final approval of the version to be published
YA	Agreement to be accountable for all aspects of the work in ensuring that questions related to the accuracy or integrity of any part of the work are appropriately investigated and resolved; final approval of the version to be published

## References

[1] Cowles RA, Berdon WE (2007). Bland-White-Garland syndrome of anomalous left coronary artery arising from the pulmonary artery (ALCAPA): a historical review. Pediatr Radiol.

[2] Bland EF, White PD, Garland J (1933). Congenital anomalies of the coronary arteries: report of an unusual case associated with cardiac hypertrophy. Am Heart J.

[3] Wilson CL, Dlabal PW, McGuire SA (1979). Surgical treatment of anomalous left coronary artery from pulmonary artery: follow-up in teenagers and adults. Am Heart J.

[4] Wesselhoeft H, Fawcett JS, Johnson AL (1968). Anomalous origin of the left coronary artery from the pulmonary trunk: its clinical spectrum, pathology, and pathophysiology, based on review of 140 cases with seven further cases. Circulation.

[5] Bunton R, Jonas RA, Lang P, Rein AJ, Castaneda AR (1987). Anomalous origin of the left coronary artery from pulmonary artery. Ligation versus establishment of a two-coronary system. J Thorac Cardiovasc Surg.

[6] Kudumula V, Mehta C, Stumper O, Desai T, Chikermane A, Miller P (2014). Twenty-year outcome of anomalous origin of left coronary artery from pulmonary artery: management of mitral regurgitation. Ann Thorac Surg.

[7] Sarioglu T, Yalçinbas Y, Erek E, Arnaz A, Türkekul Y, Avsar MK (2013). Anomalous left coronary artery originating from pulmonary artery: recovery of left ventricular function after dual coronary system restoration and clinical results. Turk J Thorac Cardiovasc Sur.

[8] Chandra MP, Debabrata B, Ashish G, Sudama T (2015). ALCAPA presenting as acute coronary syndrome in an adult: an interesting case report with short review of literature. J Cardiovasc Dis Res.

[9] Ginde S, Earing MG, Bartz PJ, Cava JR, Tweddell JS (2012). Late complications after Takeuchi repair of anomalous left coronary artery from the pulmonary artery: case series and review of literature. Pediatr Cardiol.

[10] Yau JM, Singh R, Halpern EJ, Fischman D (2011). Anomalous origin of the left coronary artery from the pulmonary artery in adults: a comprehensive review of 151 adult cases and a new diagnosis in a 53-year-old woman. Clinic Cardiol.

[11] Sabiston DC Jr, Neill CA, Taussig HB (1960). The direction of blood flow in anomalous left coronary artery arising from the pulmonary artery. Circulation.

[12] Neches WH, Mathews RA, Park SC, Lenox CC, Zuberbuhler JR, Siewers RD (1974). Anomalous origin of the left coronary artery from the pulmonary artery. A new method of surgical repair. Circulation.

[13] Takeuchi S, Imamura H, Katsumoto K, Hayashi I, Katohgi T, Yozu R (1979). New surgical method for repair of anomalous left coronary artery from pulmonary artery. J Thorac Cardiovasc Surg.

[14] Neumann A, Sarikouch S, Bobylev D, Meschenmoser L, Breymann T, Westhoff-Bleck M (2017). Long-term results after repair of anomalous origin of left coronary artery from the pulmonary artery: Takeuchi repair versus coronary transfer. Eur J Cardiothorac Surg.

[15] Dodge-Khatami A, Mavroudis C, Backer CL (2002). Anomalous origin of the left coronary artery from the pulmonary artery: collective review of surgical therapy. Ann Thorac Surg.

[16] Jonas RA (2004). Anomalies of coronary arteries. In: Jonas RA, editor. Comprehensive surgical management of congenital heart disease.

[17] Arciniegas E, Farooki ZQ, Hakimi M, Green EW (1980). Management of anomalous left coronary artery from the pulmonary artery. Circulation.

[18] Schwartz ML, Jonas RA, Colan SD (1997). Anomalous origin of left coronary artery from pulmonary artery: recovery of left ventricular function after dual coronary repair. J Am Coll Cardiol.

[19] Amanullah MM, Hamilton JR, Hasan A (2001). Anomalous left coronary artery from the pulmonary artery: creating an autogenous arterial conduit for aortic implantation. Eur J Cardiothorac Surg.

[20] Azakie A, Russell JL, McCrindle BW, Van Arsdell GS, Benson LN, Coles JG (2003). Anatomic repair of anomalous left coronary artery from the pulmonary artery by aortic reimplantation: early survival, patterns of ventricular recovery and late outcome. Ann Thorac Surg.

[21] Stern H, Sauer U, Locher D, Bauer R, Meisner H, Sebening F (1993). Left ventricular function assessed with echocardiography and myocardial perfusion assessed with scintigraphy under dipyridamole stress in pediatric patients after repair for anomalous origin of the left coronary artery from the pulmonary artery. J Thorac Cardiovasc Surg.

[22] Ben Ali W, Metton O, Roubertie F, Pouard P, Sidi D, Raisky O (2009). Anomalous origin of the left coronary artery from the pulmonary artery: late results with special attention to the mitral valve. Eur J Cardiothorac Surg.

[23] Cabrera AG, Chen DW, Pignatelli RH, Khan MS, Jeewa A, Mery CM (2015). Outcomes of anomalous left coronary artery from pulmonary artery repair: beyond normal function. Ann Thorac Surg.

[24] Alexi-Meskishvili V, Nasseri BA, Nordmeyer S, Schmitt B, Weng YG, Böttcher W (2011). Repair of anomalous origin of the left coronary artery from the pulmonary artery in infants and children. J Thorac Cardiovasc Surg.

